# Mirabegron and solifenacin are effective for the management of the increased urinary frequency induced by psychological stress in female mice

**DOI:** 10.1038/s41598-022-16487-7

**Published:** 2022-07-20

**Authors:** Eliza G. West, Catherine McDermott, Russ Chess-Williams, Donna J. Sellers

**Affiliations:** Centre for Urology Research, Faculty of Health Sciences and Medicine, Bond University, Robina, QLD 4229 Australia

**Keywords:** Bladder, Urological manifestations, Physiology

## Abstract

Evidence to support the effectiveness of β3-adrenoceptor agonist mirabegron and anti-muscarinic solifenacin in the management of bladder dysfunction caused by psychological stress is lacking. This study investigates whether mirabegron or solifenacin reduces the bladder overactivity caused by water avoidance stress (WAS) in mice. Female mice were exposed to WAS for 1 h/day for 10 days and received either placebo, solifenacin or mirabegron in drinking water. Controls were age-matched without stress exposure. Voiding behaviour and functional isolated whole bladder responses during distension and in response to pharmacological agents and electrical field stimulation was investigated. Urinary frequency was significantly increased following stress. Mice treated with mirabegron or solifenacin displayed significantly fewer voiding events compared to the stressed mice, and voiding frequency in drug-treated animals was comparable to unstressed controls. The maximal contractile responses of bladders to carbachol were significantly enhanced by stress and reduced by mirabegron but not solifenacin. The frequency of phasic bladder contractions following stimulation with carbachol was significantly enhanced following stress and remained elevated in the mirabegron treated group. However, treatment with solifenacin significantly reduced the frequency of phasic contractions to unstressed control levels. Solifenacin and mirabegron are beneficial in reducing the overall voiding dysfunction caused by WAS in mice.

## Introduction

Overactive bladder (OAB) is defined by the International Continence Society as urinary urgency, with or without urge incontinence, usually accompanied by frequency and nocturia, in the absence of urinary tract infection or other obvious pathology^1^. While many instances of OAB are idiopathic, there is evidence from both clinical and experimental studies that psychological stress can cause or exacerbate symptoms of bladder overactivity^2–4^. However, evidence as to whether the currently used clinical treatments for OAB would be useful in this patient group is lacking. The literature consistently reports that chronic water avoidance stress exposure induces an overactive phenotype in female rodents, with urinary frequency linked to central and peripheral changes^5–7^.

Antimuscarinic agents are the most common oral pharmacotherapy used for treating OAB^8^. Their main mode of action being to relax detrusor smooth muscle, but they also reduce sensory symptoms during the storage phase of the micturition cycle^9^. In the 1970s, the non-selective muscarinic antagonist oxybutynin became available, with clinical trials showing the drug was effective in managing the symptoms of OAB. However, oxybutynin is associated with numerous side-effects including dry mouth, constipation and blurred vision, leading to many patients discontinuing treatment^10,11^. Since the 1990’s more selective muscarinic antagonists were developed with the aim of improving efficacy and reducing side-effects. Solifenacin, a competitive M_3_ muscarinic receptor-selective antagonist, was found to be more selective for muscarinic effects in the bladder than in the salivary glands in in vivo and in vitro studies^12^. Whilst there is evidence that some antimuscarinic agents targeting M_1_ receptors cause cognitive impairments because they cross the blood brain barrier^13^, studies of M_3_ receptor selective drugs, such as darifenacin and tolterodine, have found that they do not cross the blood brain barrier to any significant extent and so do not cause cognitive dysfunction^13^. Since solifenacin targets M_3_ receptors, cognitive dysfunction as an adverse effect is also unlikely with this agent. Overall, tolerability of solifenacin is reported to be 85%, with efficacy of 74% after long term treatment^14^.

More recently, the β_3_-adrenoceptor (β_3_-adrenoceptor) agonist mirabegron was introduced and has shown similar efficacy to the antimuscarinics, with lower incidence of side effects as one of the most selective OAB treatments^15,16^. On a molecular level, mirabegron activates β_3_-adrenoceptors, which are coupled to Gs linked G-proteins, which in turn increase intracellular cyclic AMP (cAMP) levels, thereby activating potassium channels and causing hyperpolarisation of detrusor smooth muscle^17^. There is also some evidence that mirabegron acts to inhibit spontaneous activity in the bladder by inhibiting microcontractions which appear to be myogenic in origin^18^. Studies have also shown that the β_3_-adrenoceptor agonist causes inhibition of C- and Aδ-afferent nerves in the rat bladder^19^. Both C- and Aδ-afferent nerves convey sensations of bladder filling and a study found that mirabegron inhibited afferent activities of both fibres^18^, and this may contribute to its therapeutic benefit in patients with OAB.

Whilst solifenacin and mirabegron are effective for symptoms of OAB, it is currently unclear whether these drugs are useful for managing voiding dysfunction caused by psychological stress. This study investigates the potential benefit of the muscarinic antagonist solifenacin and the β_3_-adrenoceptor agonist mirabegron in treating the increased urinary frequency observed with water avoidance stress in adult female mice.

## Results

### Animal parameters and voiding

Animal body weight and water consumption were measured during the stress exposure period and were unchanged across all animal groups compared to the control. Bladder weight was measured after whole bladder preparations and was similarly unchanged across the groups (data not shown).

**Figure 1:**
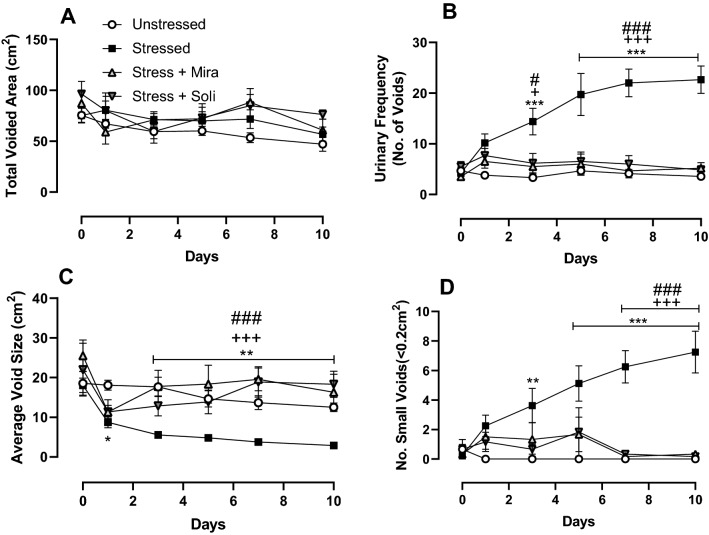
Analysis of voiding behaviour in unstressed, stressed, stress + mirabegron and stress + solifenacin mice measured as (**A**) total voided area, (**B**) urinary frequency, (**C**) average void size and (**D**) number of small voids. Data is presented as mean ± SEM (n = 6) and was analysed using two-way ANOVA with two-way repeated measures ANOVA (**p < 0.01, ***p < 0.001, Unstressed vs Stressed), (^+^p < 0.05, ^+ ++^p < 0.001 Stressed vs Stress + Mirabegron), (*p < 0.05, ^###^p < 0.01 Stressed vs Stress + Solifenacin).

The impact of stress and treatment with mirabegron or solifenacin on voiding behaviour was assessed using voiding pattern analysis. Total voided area was unchanged across the groups (Fig. 1A) indicating no change in urine production following stress or drug treatment, however urinary frequency and the number of small voids was increased with stress, while average void size was decreased with stress, with all voiding parameters significantly changed relative to the stressed group and comparable to unstressed controls in the solifenacin and mirabegron treated animals (Fig. 1B–D).

**Figure 2:**
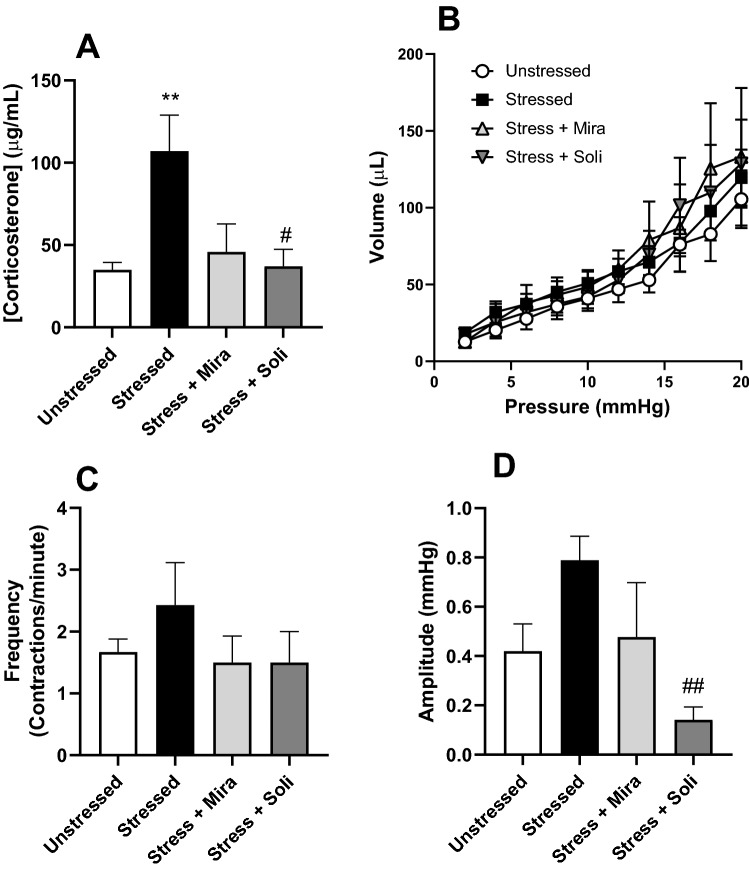
Effects of water avoidance stress or treatment of stressed mice with mirabegron or solifenacin on (**A**) plasma corticosterone, (**B**) the volume pressure relationship, and spontaneous phasic contractions in isolated bladders measured as (**C**) frequency and (**D**) amplitude. Data is presented as mean ± SEM (n = 6) and was analysed using one-way ANOVA (**A**), two-way ANOVA (**B**) with Tukey multiple comparisons test or Kruskal–Wallis with Dunn’s multiple comparisons test (**C,D**) (**p < 0.01 vs Unstressed; ^#^p < 0.05, ^##^p < 0.01 vs Stressed).

Blood samples were taken at the time of euthanasia and plasma corticosterone levels were measured. There was a significant increase in plasma corticosterone levels in the stressed group to 107 ± 21.90 µg/mL, compared to the control group, 34.9 ± 4.47 µg/mL. Solifenacin significantly decreased plasma corticosterone levels to 37.01 ± 10.45 µg/mL, and although not statistically different to the stressed group, mirabegron treatment also decreased corticosterone levels to 45.75 ± 17.03 µg/mL (p = 0.054) (Fig. 2A).

The volume-pressure relationship of isolated whole bladders during distension to filling revealed that bladder compliance was not significantly changed by stress, or treatment with mirabegron or solifenacin (Fig. 2B). Spontaneous phasic activity was evident in all isolated whole bladders from the unstressed and stressed animals during accommodation following distension to 20 mmHg, however was only evident in 83% of bladders from mirabegron treated animals and 67% of bladders from the solifenacin treated group. While the frequency of these contractions remaining unchanged across all animal groups (Fig. 2C), solifenacin treatment significantly decreased the amplitude of spontaneous phasic contractions relative to the stressed animals (Fig. 2D).

Isolated whole bladder contractile responses to KCl were measured to assess non-receptor mediated bladder contractility. While pressure responses to KCl were greater in the stressed and drug-treated animals, this was not statistically significant. KCl responses in bladders from unstressed, stressed, mirabegron and solifenacin treated animals were 30.1 ± 2.31 mmHg, 38.2 ± 4.65 mmHg, 36.92 ± 8.79 mmHg, and 38.5 ± 5.28 mmHg, respectively.

**Figure 3:**
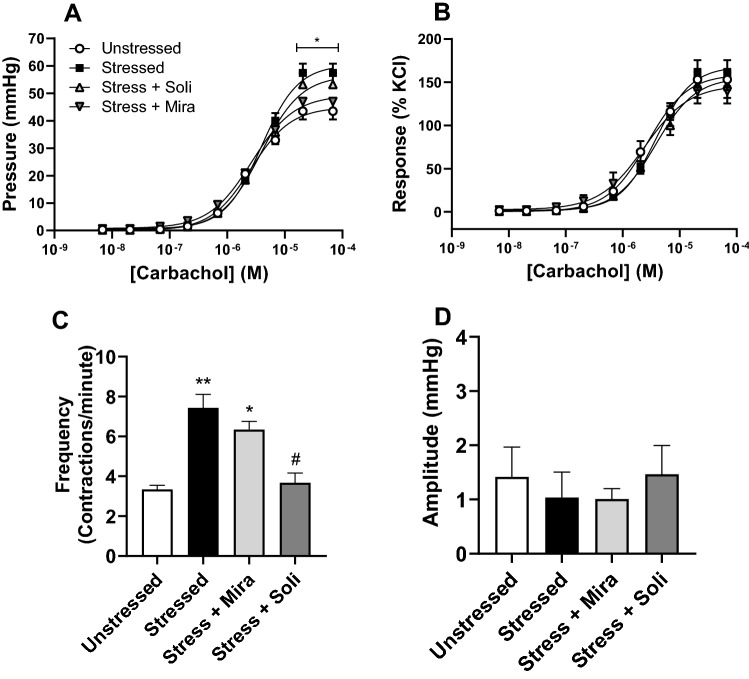
Effect of water avoidance stress or stress plus treatment with mirabegron or solifenacin on cumulative carbachol concentrations measured as (**A**) change in pressure and (**B**) normalised to KCl response; and phasic response to 1 µM carbachol measured as (**C**) frequency and (**D**) amplitude of phasic contractions. Data represents mean ± SEM (n = 6) and was analysed using two-way ANOVA with Tukey multiple comparisons test (**A,B**) or Kruskal–Wallis test Dunn’s multiple comparisons test (**C,D**) (**A**: *p < 0.05 Stressed vs Unstressed; **C**: *p < 0.05, **p < 0.01 Stressed vs Unstressed; ^#^p < 0.05 Stress + Solifenacin vs Stressed).

Bladder contractile responses to cholinergic stimulation with cumulative carbachol concentrations, were concentration dependent, with no significant difference in the pEC50 values observed (Table 1, Fig. 3A). However, the maximal contractile response to carbachol was significantly enhanced following 10-days stress exposure (44.9 ± 1.03 mmHg Unstressed vs 60.3 ± 2.18 mmHg Stressed (p < 0.01)) and remained significantly elevated in the solifenacin treated group (56.5 ± 2.63 mmHg (p < 0.05)) relative to unstressed controls. Maximal responses to carbachol were however significantly decreased by mirabegron treatment (49.2 ± 3.98 mmHg (p < 0.05) relative to the stressed group. When the response to carbachol was expressed as a percentage of the KCl response, no significant differences were observed in the maximal responses between the groups (Table 1; Fig. 3B). The phasic component of the carbachol-induced (1 µM) contractile response was also quantified, and the frequency of contractions was significantly enhanced in the stressed and mirabegron treated animals compared to unstressed controls (Fig. 3C). Treatment with solifenacin however, significantly reduced the frequency of phasic contractions to control levels (Fig. 3C). The amplitude of the phasic contractions remained unchanged across all groups (Fig. 3D).

**Table 1:** Isolated whole bladder responses to carbachol and isoprenaline in unstressed, stressed, stress + mirabegron and stress + solifenacin groups.

	Unstressed	Stressed	Stress + Mirabegron	Stress + Solifenacin
**Carbachol**				
pEC_50_	5.6 ± 0.03	5.4 ± 0.05	5.6 ± 0.12	5.4 ± 0.06
Maximal response ∆Pressure (mmHg)	44.9 ± 1.03	60.3 ± 2.18**	49.2 ± 3.98 **#**	56.5 ± 2.63*
Response (% KCl)	159 ± 7.16	168 ± 9.43	146 ± 11.6	155 ± 9.71
**Isoprenaline**				
pEC_50_	6.7 ± 0.18	6.8 ± 0.13	6.9 ± 0.19	6.9 ± 0.10
Maximal response (% of pre-contraction)	91.7 ± 6.11	67.5 ± 3.30 **	62.5 ± 4.18***	73.1 ± 2.77*

Data is presented as mean ± SEM (n = 6) analysed using one-way ANOVA with Tukey–Kramer multiple comparisons test (*p < 0.05, **p < 0.01, ***p < 0.001 vs Unstressed; # p < 0.05 vs Stressed).

Isolated whole bladder responses to the purinergic agonist ATP were quantified and while an increased response was evident in the Stressed group (11.9 ± 1.01 mmHg) compared to the Unstressed group (8.5 ± 1.3 mmHg) this was not statistically significant. The intravesical pressure response to ATP in bladders from mirabegron and solifenacin treated animals was 11.2 ± 0.91 mmHg and 8.96 ± 1.38 mmHg respectively.

**Figure 4:**
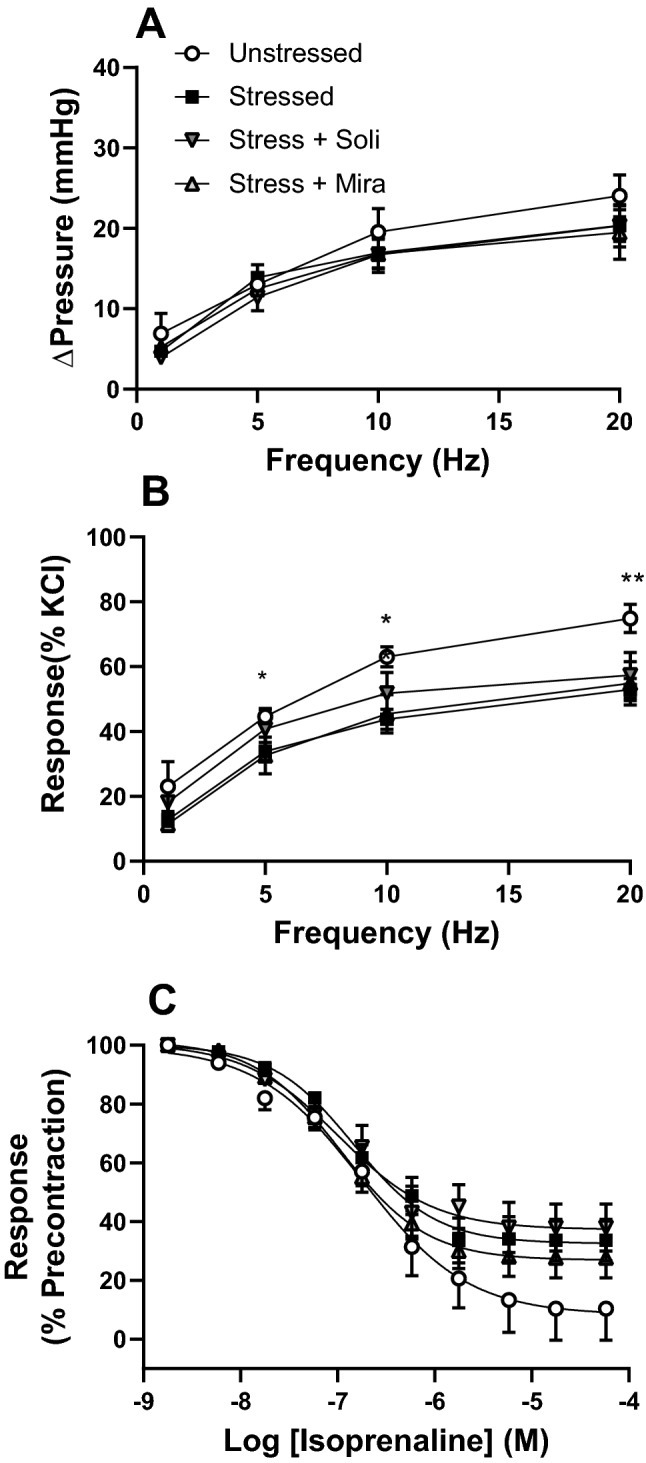
Responses of isolated whole bladders from unstressed, stressed, and mirabegron and solifenacin treated mice to electrical field stimulation measured as (**A**) change in intravesical pressure and (**B**) normalised to KCl response; and (**C**) cumulative concentrations of the beta-adrenoceptor agonist isoprenaline following carbachol pre-contraction (1 µM). Data represent mean ± SEM (n = 6) and was analysed using two-way ANOVA with Tukey multiple comparisons test (*p < 0.05, **p < 0.01 Unstressed vs Stressed).

Nerve mediated responses were assessed using EFS at 1, 5, 10 and 20 Hz. Stress did not change the intravesical pressure response to electrical field stimulation nor did treatment with mirabegron or solifenacin (Fig. 4A). However, when the response to EFS was normalised to KCl contraction, the response in the Stress group was significant decreased compared to Unstressed controls, at 5-20 Hz (Fig. 4B). Relative contribution of ACh and ATP to nerve mediated responses was assessed at 20 Hz, with addition of pharmacological agents. The muscarinic antagonist atropine reduced the response to EFS by 16.9 ± 2.6% in unstressed controls, and this was not altered by stress (15.4 ± 2.7%) or drug treatment (9.6 ± 1.7% for mirabegron; 14.9 ± 2.4% for solifenacin). The addition of αβmATP to desensitise the P_2_X_1_ purinoceptors further decreased the EFS response by 43.8 ± 4.53% in unstressed controls, and this was similar in stressed animals (51.5 ± 3.2%) and drug treated animals (46.4 ± 6.0% for mirabegron; 50.7 ± 8.2% for solifenacin).

Cumulative additions of the beta-adrenoceptor agonist isoprenaline following carbachol pre-contraction revealed concentration dependent bladder relaxation (Fig. 4C). No significant difference in pEC50 was observed, however the maximal relaxation response was significantly less in all three stress groups relative to unstressed controls (Table 1).

**Figure 5:**
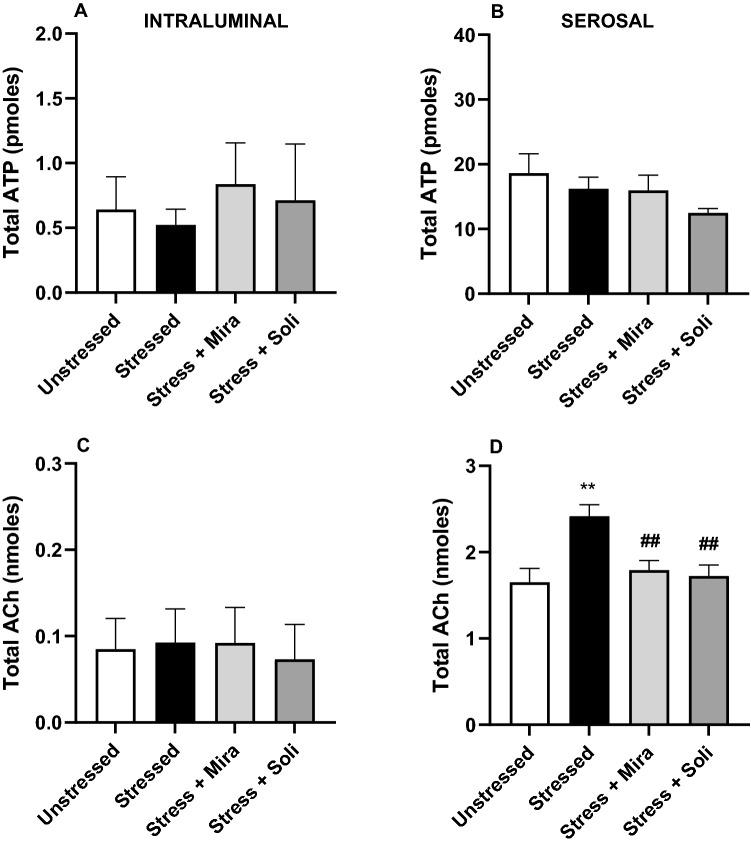
Effect of water avoidance stress or stress and treatment with mirabegron or solifenacin on total release of (**A,B**) ATP and (**C,D**) ACh into the intraluminal fluid (from inside of the bladder lumen) and serosal fluid (from the bathing solution) following distension of isolated whole bladders to 20 mmHg. Data is presented as mean ± SEM (n = 6) and was analysed using one-way ANOVA with Tukey–Kramer multiple comparisons test (**p < 0.01 vs Unstressed; ^##^p < 0.01 vs Stressed).

Release of urothelial ATP and ACh into the intraluminal and serosal fluid when isolated bladders were distended to 20 mmHg was assessed to determine the impact of stress and drug treatment on signalling mediator release. While neither stress nor drug treatment significantly affected ATP release or intraluminal ACh release (Fig. 5A–C); total ACh in the serosal fluid was significantly elevated following water avoidance stress exposure (Fig. 5D). Treatment with mirabegron or solifenacin significantly reduced release of ACh into the serosal fluid to unstressed levels.

## Discussion

Increased urinary frequency is common among individuals experiencing chronic psychological stress^4,20^. Similarly, repeated water avoidance stress causes an overactive voiding phenotype in mice, which is partially reduced by the anxiolytic sertraline^2,5,21^. Water avoidance stress has long been reported to affect bladder function via alterations to both central and peripheral processes. Visceral hypersensitivity and increased bladder afferent nerve activity during bladder filling has been reported in rodents following water avoidance stress exposure, as well as increased engagement in central micturition circuits^5,6^. Bladder frequency associated with water avoidance stress is also linked to lymphocytic and mast cell infiltration in the bladder wall^22^, increased contractile responses^7^ and increased cyclopxygenase-2 expression in the detrusor^23^.

In this study, mirabegron and solifenacin significantly decreased stress-induced voiding frequency. The decreased voiding frequency was accompanied by increased average void size and decreased number of small voids. There was no difference in total voided area, indicating that the mice were producing the same volume of urine and therefore the changes observed were in voiding phenotype rather than urine production. Both drugs returned voiding behaviour to control levels despite continued stress exposure.

Overactive bladder symptoms correlate with increased anxiety and depression in patients. While there is some evidence that both mirabegron and solifenacin can reduce the associated symptoms of anxiety and depression in clinical studies^24,25^, here we observed that oral treatment with solifenacin or mirabegron can reduce the hormonal stress response associated with water avoidance stress. Adrenocorticotropic hormone is a regulator of steroidogenesis in the adrenal cortex; however, it has been suggested that there are other local and central mechanisms controlling adrenocortical steroidogenesis, including corticosterone release. Muscarinic receptors regulate numerous central and peripheral functions, and the HPA axis in humans and rodents is reportedly affected by activation or blockade of these receptors^26^. Central muscarinic receptors reportedly modulate corticosterone secretion, with some conflicting evidence of the specific receptor subtype involved (as reviewed by Thomsen, et al.^27^). A recent study using wild-type, M_2_ and M_4_ single-knockout mice reported that administration of a muscarinic agonist stimulates corticosterone release in vivo via M_2_ muscarinic receptors^28^. Direct actions of acetylcholine on adrenal cortical cells to simulate steroidogenesis was first reported in 1977^29^ and later confirmed to rely on activation of muscarinic receptors in several species^30,31^. The presence of cholinergic fibres in the adrenal cortex has also been observed in several species^32^. This would suggest that the anti-muscarinic solifenacin may reduce corticosterone secretion via actions on central or adrenocortical muscarinic receptors.

Stress-induced increase in plasma corticosterone in rats is also influenced by β-adrenoceptors in the hypothalamic paraventricular nucleus^33^. Additionally, catecholaminergic nerves are distributed in the subcapsular regions of the adrenal gland including the zona fasciculata^34,35^, and noradrenaline may be an important regulator of glucocorticoid secretion, possibly via β1-adrenoceptors^36^. These provide a plausible mechanism by which mirabegron reduced plasma corticosterone in stressed mice in the current study. Mirabegron acts specifically on β_3_-adrenoceptors which are expressed in several different tissues, including the hippocampus, amygdala, hypothalamus and cerebral cortex^37–39^; with growing interest in targeting these receptor subtypes to treat disorders such as anxiety and depression^37^. Acute administration of the β_3_-adrenoceptor agonist CL 316243 increased hypothalamic 5-HT synthesis^40^. A study by Stemmelin, et al.^41^ used social defeat and forced swim test to measure anxiety and depression related behaviour in rats. Treatment with the β_3_ agonist, SR58611A (amibegron), resulted in anxiolytic and antidepressant behavioural effects^41^. The study also found that the β_3_ agonist increased synthesis of 5-HT and tryptophan in the cortex, hippocampus, and hypothalamus which correlates with other studies^42^. Several links have been made between 5-HT and cortisol/corticosterone release which indicate that 5-HT may influence the peripheral adrenomedullary response to increase cortisol in humans and corticosterone levels in rodents^43–45^. The data from the present study suggests that the therapeutic benefit of mirabegron and solifenacin in managing water avoidance stress induced voiding dysfunction may be by inhibiting stress induced activation of the HPA axis and therefore downstream targets, rather than just direct effects on the bladder itself.

Stretch of the urothelium promotes release of mediators, including ACh and ATP, which play an important role in normal bladder function^46^. While ATP release was unchanged in the present study, ACh release in the serosal fluid was significantly increased in bladders from stressed animals, and significantly decreased to unstressed levels after mirabegron and solifenacin treatment. Release of urothelial ACh is reportedly mediated by the organic cation transporters OCT1 and OCT3, which are involved in non-vesicular release of ACh from the urothelium^47^. While it is unlikely that solifenacin and mirabegron act directly to inhibit non-neuronal ACh release from the urothelium, there is some evidence from an in vitro study that shows mirabegron to weakly inhibitOCT1 in mouse kidney-derived S2 cells, with an IC_50_ of 47.2 µM (unpublished data reported by Groen-Wijnberg, et al.^48^). Interestingly, this concentration is 175-fold higher than the mean maximum plasma concentration in healthy subjects after the administration of mirabegron at 50 mg once a day, the highest approved dose^49^. There is, however, no evidence that solifenacin has any effect on these transporters. Multiple studies have identified that the urothelium expresses a range of muscarinic receptors^50,51^ and beta-adrenoceptors^52,53^. However, it is still unclear whether these receptors regulate non-neuronal ACh release. It is possible that the effects on ACh release was due to the decreased hormonal stress response caused by mirabegron and solifenacin, rather than a direct effect at urothelial receptors.

While voiding frequency was reduced by both mirabegron and solifenacin treatment, the increase in bladder contractility observed following stress was only reduced by mirabegron. This suggests that the increased contractility does not play a causal role in the voiding dysfunction observed with stress and may instead be a local compensatory mechanism in response to stress. An in vitro study on mouse urinary bladder reported that solifenacin does not alter the maximal response to carbachol^54^, unlike darifenacin. Similarly*,* solifenacin increases bladder capacity without affecting maximum micturition pressure in rodents in vivo^55,56^ The enhanced contractile response caused by repeated WAS may involve calcium-sensitization through the Rho-kinase pathway. Since activation of β_3_-adrenoceptors leads to increased levels of cAMP, known to inhibit the Rho-kinase pathway in detrusor smooth muscle^57^, this may explain the benefit observed with mirabegron.

Relaxation of human detrusor is mediated predominately by β_3_-adrenoceptors^58^, whilst involvement of both β_2_ and β_3_-adrenoceptors has been reported in rodents^59^. The present study found that maximal relaxation of isolated whole bladders to isoprenaline was significantly reduced following stress exposure and neither mirabegron nor solifenacin treatment affected this. While not assessed in the current study, this change may reflect decreased receptor density or desensitization of β-adrenoceptors in the mouse bladder due to chronic activation of the stress response as has been reported in the literature. Chronic stress in caregivers has been linked to reduced β_2_-adrenoceptor sensitivity and density in immune cells compared to non-stressed counterparts^60^. Prolonged exposure to endogenous or exogenous catecholamines reduces the physiological response to β-adrenoceptor stimulation, including depressed bronchodilatory responses in airways^61^. Previous research suggests that β_3_-adrenoceptors are more resistant to desensitization than β_1_- and β_2_-adrenoceptors^62,63^, however given the role of β_2_-adrenoceptors in the rodent bladder the decreased maximal response with stress may reflect a change in this specific sub-type. Regardless of the cause, the altered bladder relaxation response does not appear to play a causal role in voiding dysfunction, given that mice in the drug treated groups had normal voiding behaviour despite depressed maximal relaxation.

Phasic activity occurs in the normal bladder, although it’s role in micturition and cellular origin is not yet clear. Spontaneous phasic contractions may be responsible for increased pressure during bladder filling and may evoke afferent nerve firing which could be linked with urinary urgency^64^. The frequency of the phasic activity induced by muscarinic stimulation was significantly enhanced by stress and reduced by solifenacin treatment. Non-voiding activity is reportedly sensitive to muscarinic antagonists and β_3_-adrenoceptor agonists. Cystometry in conscious rats with replicated partial bladder outflow obstruction showed that as the bladder filled intravenous mirabegron and tolterodine reduced large non-voiding contractions, mirabegron only affecting frequency but tolterodine affecting both frequency and amplitude^65^. In rats mirabegron inhibits Aδ-fibre mechano-sensitive afferent activity, decreasing afferent signalling from the periphery to the brain^18^. Thus, solifenacin may be acting to decrease spontaneous activity while mirabegron may decrease afferent signalling, contributing to the therapeutic effect and leading to decreased urgency to void and decreased urinary frequency in the treated mice.

Treatment with the muscarinic antagonist, solifenacin, and β-adrenoceptor agonist, mirabegron, is effective in reducing the effects of water avoidance stress on voiding behaviour. Both therapeutics also reduced stress-induced changes in urothelial ACh release and plasma corticosterone levels. The anxiolytic sertraline tested previously was less effective than both mirabegron and solifenacin in managing voiding changes caused by water avoidance stress, with voiding remaining elevated compared to unstressed animals^21^, suggesting that the muscarinic antagonist and β-adrenoceptor agonist may be more promising to test clinically. These results indicate that management of bladder dysfunction caused by psychological stress may benefit from the addition of an antimuscarinic such as solifenacin or a β_3_-adrenoceptor agonist such as mirabegron.

## Methods

### Murine stress model and drug treatment

The study is reported in accordance with ARRIVE guidelines (https://arriveguidelines.org). All procedures were performed in accordance with the Australian Code for the Care and Use of Animals for Scientific Purposes and with the approval of the University of Queensland Animal Ethics Committee. Adult female C57Bl/6J (12–14 weeks in age; n = 6 in each group) from Animal Resources Centre (Western Australia) were used in this study, and housed in pairs under environmentally controlled conditions, with 12-h light–dark cycles, with access to food and water ab libitum. Estrous cycle stage was not assessed. Mice were randomly allocated into four experimental groups: (1) Unstressed, (2) Stressed, (3) Stress + Mirabegron and (4) Stress + Solifenacin.

Water avoidance stress (WAS) is commonly used in rodents to induce a stress response and was performed as previously described^7^. Briefly, mice in the stress groups were placed individually on a pedestal surrounded by water for 1 h/day for 10 consecutive days. After each stress exposure, mice were returned to their normal housing. The unstressed group consisted of age-matched control mice housed under normal conditions and not exposed to water avoidance stress protocols.

The adult human daily dose for mirabegron (10 mg/day) and solifenacin (50 mg/day) was used to calculate the equivalent dose in mice (2 mg/kg/day for mirabegron and 10 mg/kg/day for solifenacin) based on a published dose conversion guide for humans to animals^66^. Both drugs were prepared in an oral suspension (National Custom Compounding, QLD) and added to the drinking water of animals with the final concentration of solifenacin being 50 µg/ml and mirabegron being 10 µg/ml. Mice in the unstressed and stressed groups received drug-free suspension (vehicle) in their drinking water. Animals received mirabegron, solifenacin or vehicle in their drinking water during the 10-day experimental protocol. Water consumption was assessed and no significant difference in volume consumed was observed between groups (Data not shown).

### Voiding pattern analysis

Voiding behaviour was assessed as previously described to determine how stress and stress with mirabegron or solifenacin treatment affects urinary frequency, total voided volume, average void size and number of small voids, compared to unstressed/control animals^7^. Voiding pattern analysis (VPA) was performed at baseline (Day 0) and at intervals (1, 3, 5, 7 and 10 days) during the WAS protocol. At the beginning of the light cycle mice were placed individually for 4 h, in cages lined with hardened ashless filter paper (Filtech; Quantitative 2um grade 225). Animals had free access to food and drinking water during this time. Filter papers were collected, and urine spots detected using a Molecular Imager ChemiDoc XRS ultraviolet transilluminator (BioRad, California USA). The papers were photographed, digitized, and then analysed using Image J software.

### Isolated whole bladder preparation

Isolated whole bladders were used for functional bladder studies and set up as previously described^67,68^. Twenty-four hours following the final water avoidance stress exposure, mice were euthanized by cervical dislocation, the bladder was isolated, and a three-way catheter was inserted through the urethra into the bladder. The urethra and ureters were ligated, and the bladder was placed into a bath of gassed (95%O_2_/5% CO_2_) Krebs-bicarbonate solution (composition in mM: NaCl 118, NaHCO_3_ 24.9, CaCl_2_ 1.9, MgSO_4_ 1.15, KCl 4.7, KH_2_PO_4_ 1.15, and d-glucose 11.7) at 37 °C. The three-way catheter was attached to an infusion pump to allow bladder filling, a pressure transducer to record intravesical pressure, and an outflow syringe to collect intraluminal fluid and allow bladder emptying. Intravesical pressure was measured using a pressure transducer (GlobalTown Microtech, Sarasota, FL) connected to a PC via a PowerLab data acquisition system (AD Instruments, Sydney, Australia), using LabChart 7 software (AD Instruments). Following equilibration, bladder distensions were performed by intravesical infusion of saline at a rate of 30 µL/min up to a maximum pressure of 40 mmHg to assess viability, and to 20 mmHg for all further distensions.

To determine the effect of water avoidance stress and stress with mirabegron or solifenacin treatment on urothelial mediator release, intraluminal fluid was collected from inside of the bladder lumen via the catheter following distension to 20 mmHg (Intraluminal volume: Unstressed 105 ± 18.8µL; Stressed 119 ± 18.3µL; Stress + Mirabegron 133 ± 44.8µL; Stress + Solifenacin 129 ± 28.7µL;), in addition to a 200µL sample of serosal fluid from the bathing solution (8 mL). Samples were stored at −80 °C until analysis of ATP and acetylcholine (ACh) levels. Quantification of ATP and ACh was carried out using the ATP Determination Kit (Molecular Probes), and the Acetylcholine Amplex Red Assay Kit (Molecular Probes) respectively. The assays were performed according to manufacturer instructions, with luminescence and fluorescence (excitation 540, emission 590 nm) measured, using a Modulus micro-plate reader (Promega).

Following bladder distension to 20 mmHg, bladders were allowed to equilibrate/accommodate for approximately 60 min, during which time spontaneous phasic activity was measured as (1) the frequency of spontaneous contractions per minute and (2) the amplitude measured as the change in intravesical pressure from the trough to the peak of the contractions.

The effect of stress and stress with mirabegron or solifenacin treatment on nerve-evoked contractile bladder responses was assessed by electric field stimulation (EFS). The bladder was electrically stimulated (0.1 ms pulse-width, 50 V) for 5 s, every 100 s at 1–20 Hz. Bladders were stimulated at each frequency until 3 consistent responses were obtained and contractions were measured as the increase in intravesical pressure from baseline. EFS was repeated at 20 Hz in the absence and presence of atropine (1 µM) to block muscarinic receptors and αβ-methylene ATP (αβmATP, 10 µM, twice) to desensitize P2X receptors and thus remove cholinergic and purinergic components, respectively. Application of tetrodotoxin (0.1 µM), abolished responses to EFS, confirming the neurogenic origins of the pressure responses observed.

Intravesical pressure responses to pharmacological agents were also assessed by addition of cumulative concentrations of the muscarinic agonist carbachol, the purinergic agonist ATP (10 mM) and relaxations to the β-adrenoceptor agonist isoprenaline assessed following precontraction with carbachol (1 µM). Non-receptor mediated contractile bladder responses were also assessed using KCl (60 mM). All contraction and relaxation responses were measured as change in pressure from baseline.

At the time of sacrifice, a venous blood sample was taken, and plasma corticosterone levels quantified using the Corticosterone Competitive ELISA (Invitrogen) according to the manufacturer’s instructions. Blood samples were collected in the morning to avoid circadian variation in corticosterone levels.

### Data and statistical analysis

All experiments were randomized, with six mice per experimental group and each experimental protocol started on a different day. Results are expressed as mean ± standard error of the mean (SEM). Data were analysed using ordinary one-way ANOVA or repeated measures two-way ANOVA with Tukey–Kramer multiple comparisons test when data was normally distributed, or Kruskal–Wallis test with Dunn’s multiple comparisons test when data did not meet normality. All analysis was performed using GraphPad Prism version 8 software (GraphPad, San Diego, CA). Significance levels were defined as P < 0.05.

### Ethics approval

The work in this study was performed in accordance with the Australian Code for the Care and Use of Animals for Scientific Purposes and with the approval of the University of Queensland Animal Ethics Committee (BOND/536/17).

## Data Availability

The datasets generated during and/or analysed during the current study are available from the corresponding author on reasonable request.
